# Characterization of dairy cow rumen bacterial and archaeal communities associated with grass silage and maize silage based diets

**DOI:** 10.1371/journal.pone.0229887

**Published:** 2020-03-02

**Authors:** Jueeli D. Vaidya, Sanne van Gastelen, Hauke Smidt, Caroline M. Plugge, Joan E. Edwards

**Affiliations:** 1 Top Institute Food and Nutrition, Wageningen, The Netherlands; 2 Laboratory of Microbiology, Wageningen University & Research, Wageningen, The Netherlands; 3 Animal Nutrition Group, Wageningen University & Research, Wageningen, The Netherlands; The University of Sydney, AUSTRALIA

## Abstract

The objective of the present study was to characterize the rumen bacterial and archaeal communities in dairy cows fed different ratios of maize silage (MS) and grass silage (GS), and place the findings in the context of ruminal fermentation as well as previously reported methane (CH_4_) emissions. Rumen fluid from 12 rumen cannulated dairy cows was collected after 10 and 17 days of feeding one of four diets, all of which had the same roughage to concentrate ratio of 80:20 based on dry matter (DM). Roughage in the four diets (GS100, GS0, GS67, GS33) consisted of either 1000 g/kg DM GS (GS100), 1000 g/kg DM MS (GS0), or a mixture of both silages in different proportions [667 g/kg DM GS and 333 g/kg DM MS (GS67); 333 g/kg DM GS and 677 g/kg DM MS (GS33)]. Total volatile fatty acid (VFA) concentrations and the molar proportions of the ruminal VFA were not affected by diet. Only the molar proportion of isovalerate was affected by time, being lower on day 17 than on day 10. Bacterial and archaeal concentrations were not affected by diet but increased from day 10 to day 17. The bacterial community composition was affected by diet, time and diet × time, whereas the archaeal community composition was only affected by diet. Several bacterial and archaeal genus level groups were associated with diet, but not with time. Analysis indicated the increased use of hydrogen by succinate and lactate producing bacteria is likely to at least partially explain the previously reported lower CH_4_ emissions from MS fed dairy cows. Furthermore, time had a significant effect on both bacterial and archaeal concentrations, and also bacterial community composition. This indicates that the rumen microbiota had not stabilized after 10 days of feeding the experimental diets.

## Introduction

Dietary composition, geographical location, cow breed and the health of the host animal are known factors that influence the rumen microbial community structure [1,2]. Of all these factors, diet is considered to be the largest driver of change in ruminal fermentation, as well as of changes in the associated microbiota and enteric methane (CH_4_) production [3,4]. Enteric CH_4_ is produced by ruminal methanogenic archaea, contributing to 16% of the total anthropogenic global greenhouse gas emissions [5], and is the largest source of CH_4_ emissions from agriculture [5]. Decreasing CH_4_ emissions has, therefore, become a major concern in ruminant livestock production, and has been a prime research focus in recent years. Several dietary strategies appear to be effective in reducing CH_4_ emissions from dairy cattle [6].

Grass silage (GS) and maize silage (MS) represent the major components in dairy cow diets. Generally, GS has a higher fiber content (i.e., neutral detergent fiber and acid detergent fiber), whereas MS has a higher starch content. Fermentation of starch favors the ruminal production of propionate and decreases ruminal pH, which reduces hydrogen (H_2_) availability and activity of rumen methanogens and consequently enteric CH_4_ production [7–9]. Van Gastelen et al. [10] found that, compared to a GS based diet, an MS based diet quadratically decreased CH_4_ yield (g/kg DMI) from lactating dairy cows. When replacing GS completely with MS, this represented a decrease of 11% in CH_4_ yield [10]. As well as decreasing CH_4_ yield, replacing GS with MS led to an increase in the molar proportions of butyrate, whereas the total volatile fatty acid (VFA) concentration and the molar proportions of acetate and propionate were unaffected [10].

It is unclear if and how the changes reported by Van Gastelen et al. [10] in CH_4_ emissions and fermentation characteristics are related to changes in the rumen microbiota. Additionally, the majority of rumen microbial studies to date have primarily focused on starch in the context of cereal grains [2,11,12] rather than different types of roughages. Hence, the objectives of the present study were (1) to investigate the effect of replacing fiber-rich GS with starch-rich MS on the rumen bacterial and archaeal diversity and concentrations using samples collected 10 and 17 days after the introduction of the experimental diets, and (2) to place the findings in the context of ruminal fermentation as well as previously reported data on CH_4_ emission [10].

## Materials and methods

### Animals, study design and ethics statement

All experimental procedures relating to the animal experiment were in accordance with Dutch law and approved by the Animal Care and Use Committee of Wageningen University & Research. Rumen fluid (RF) was collected from 12 rumen cannulated dairy cows, which represented a subset of the 32 dairy cows used for the previous study of Van Gastelen et al. [10]. Hence, the experimental design of the present study is similar. In short, the previous study followed a completely randomized block design with four dietary treatments and 32 multiparous lactating Holstein-Friesian cows with an average milk production of 34.0 ± 5.71 kg/d and 192 ± 87 DIM at the start of the experiment. Cows were blocked in groups of four according to lactation stage, parity, milk production, and presence of a rumen cannula (12 cows), and within each block cows were randomly assigned to one of four dietary treatments. The dietary treatment period lasted 17 days and for each cow consisted of a dietary adaptation period of 12 days in a tie-stall followed by a five-day period in a climate respiration chamber (CRC) to determine CH_4_ emission. Two large CRCs were used, each containing two individual airtight compartments. The CRCs were equipped with thin walls with windows, to ensure cows could see and hear each other in order to minimize the effect of social isolation on cow behavior and performance. A detailed description of the CRC design and gas measurements is reported by van Gastelen et al. [10]. For the CH_4_ emissions, three full 24-h periods were used (i.e., starting at 0800 h of d 14 until 0800 h of d 17).

For the present study, the three blocks which contained the 12 rumen cannulated dairy cows were used for RF sampling. During the study one of the cows was diagnosed with mastitis and treated locally (i.e., in the udder directly) with antibiotics and intravenously with a painkiller. Despite this treatment, the cow was retained in the study as it was otherwise healthy and, relative to the other animals in the study, had normal feed intake, CH_4_ emissions and ruminal fermentation parameters [10].

### Dietary treatments

All dietary treatments had a roughage to concentrate ratio of 80:20 based on dry matter (DM) content. The composition of the concentrate was similar for all four treatments, whereas the roughage was GS, MS, or a mixture of both (ingredient as percentage of the total amount of roughage in the diet on a DM basis): 100% GS (**GS100**), 67% GS and 33% MS (**GS67**), 33% GS and 67% MS (**GS33**), and 100% MS (**GS0**). The ingredient and chemical composition of the four experimental diets are shown in Table 1. The cows were fed ad libitum during the first seven days of the adaptation period. From day 8 to 17, feed intake was restricted to 95% of the ad libitum DM intake (DMI) of the cow within a block consuming the lowest amount of feed during day 5 to 8.

**Table 1:** Diet ingredient and chemical composition (g/kg DM, unless otherwise stated)^#^.

Item	Diet			
	GS100	GS67	GS33	GS0
**Ingredient**				
Grass silage	800	533	267	-
Corn silage	-	267	533	800
Concentrates	200	200	200	200
**Chemical Composition**				
Organic matter	924	931	938	945
Crude protein	192	182	172	163
Crude fat	22	22	21	21
Gross energy (MJ/kg of dry matter)	18.8	18.7	18.6	18.5
Neutral detergent fiber	431	396	360	325
Acid detergent fiber	233	219	204	190
Acid detergent lignin	14	14	15	15
Starch	5	91	177	262
Sugar	130	98	66	34
Organic matter	924	931	938	945
Crude protein	192	182	172	163

^#^Adapted from Van Gastelen et al. [10].

### Rumen sampling

The RF was sampled on days 10 and 17, i.e., before and directly after the climate respiration chamber phase, respectively. The RF was collected four hours after morning feeding according to the method described by Van Zijderveld et al. [13]. The RF was obtained with a rigid polyvinyl chloride (PVC) tube, which was perforated at the end (2 mm holes) to allow the RF to enter the tube. A piece of plastic tubing was inserted into the PVC tube and by application of a vacuum, RF was aspirated in three equal volumes from the front and middle of the ventral sac and from the cranial sac of the rumen. The RF sampled from the three regions was subsequently pooled, aliquoted in ~50 mL portions, immediately frozen on dry ice, and within 2 hours of collection transported to the lab where it was stored at -80°C until DNA extraction and VFA analysis.

### Determination of ruminal VFA concentrations

The RF (1 mL) from the 12 cows at the two time points (i.e., day 10 and day 17) was centrifuged at 10,000 g for 10 min. Metabolites present in the supernatant were separated by a Spectrasystem HPLC (Thermo Scientific, Breda) equipped with a Metacarb 67H column (Agilent, 300 × 65 mm) and quantified with a Refractive Index detector. Column temperature was 45°C, and 5 mM sulfuric acid was used as eluent. Flow rate was set at 0.9 mL/min. HPLC data analysis was performed in Chromeleon 7 software (Thermo Scientific). For calibration of the machine, a standard mix of acetate (2 mM), propionate (5 mM), butyrate (5 mM), isovalerate (10 mM) and valerate (20 mM) was used. DMSO (10 mM in 0.1N H_2_SO_4_) was always included as an internal standard.

### DNA extraction

Total genomic DNA was extracted from 24 RF samples (i.e., two time points for each of the 12 animals). Prior to DNA extraction, RF samples (1 mL) were centrifuged at 15,000 g for 10 min at 4°C, and the cell pellets were used for DNA extraction as previously described [4]. Briefly, cells were lysed using repeated bead beating, and the lysate was further processed in a customized MaxWell® 16 Tissue LEV Total RNA Purification kit cartridge (XAS1220) (Promega Biotech AB, Stockholm, Sweden). The quantity and purity of the DNA in the obtained extracts was assessed using a NanoDrop ND-1000 spectrophotometer (NanoDrop® Technologies, Wilmington, DE, USA).

### qPCR analysis

Quantitative PCR (qPCR) assays targeting bacterial and archaeal 16S ribosomal RNA (rRNA) genes were performed using a BioRad CFX96 system (Bio-Rad Laboratories). The qPCR reactions were carried out in triplicate as previously described by Van Lingen et al. [4], except that the reaction volumes were 25 μl. For bacterial qPCR, the forward primer Bact1369F (5′- CGGTGAATACGTTCYCGG -3′) and the reverse primer Prok1492R (5′- GGWTACCTTGTTACGACTT -3′) were used [14]. For archaeal qPCR, the forward primer Arch787F (5′- ATTAGATACCCSBGTAGTCC -3′) and the reverse primer Arch1059R (5′- GCCATGCACCWCCTC -3′) were used [15].

For standard curve preparation, a bacterial and an archaeal 16S rRNA gene PCR product was prepared as previously described [4]. DNA concentration and amplicon size were used to calculate the number of amplicon copies, and 10-fold serial dilutions were prepared in water from 10^8^ to 10^0^ amplicon copies/μl.

### Barcoded 16S rRNA gene amplicon sequencing

For the analysis of bacterial and archaeal community composition, barcoded amplicons of the 16S rRNA genes were generated using a 2-step PCR strategy [16]. With this strategy, a sequence tag (i.e., UniTag) is added to the forward (UniTag 1) and reverse (UniTag 2) primers that are used to target the gene of interest in the first PCR step. The second PCR step is then employed to add an 8-nucleotide sample specific barcode to the UniTag primer target sequences obtained from the first amplicon, as previously described [17].

For bacterial composition profiling, in the first PCR step the forward primer 27F-DegS: 5′- GTTYGATYMTGGCTCAG -3′ [18], and the reverse primer mix of 338R–I: 5′- GCWGCCTCCCGTAGGAGT -3′ [19] and 338R–II: 5′- GCWGCCACCCGTAGGTGT -3′ [20] were used with attached UniTag1 (forward primer: 5′- GAGCCGTAGCCAGTCTGC -3′) and UniTag2 (reverse primer mix: 5′- GCCGTGACCGTGACATCG -3′) linkers, respectively [16]. For archaea composition profiling, in the first PCR step 518F (5′ - CAGCMGCCGCGGTAA -3′) [21] was used as the forward primer with UniTag1, and the reverse primer 905R (5′ - CCCGCCAATTCCTTTAAGTTC– 3′) [22] with UniTag2.

The first PCR step was performed in a total volume of 50 μL containing 10 μL 1× HF buffer (Finnzymes, Vantaa, Finland), 1 μL dNTP Mix (10 mM; Promega), 1 U of Phusion® Hot Start II High-Fidelity DNA polymerase (2 U/μL) (Finnzymes), 500 nM each of the primers UniTag1-27f Deg S and UniTag2-338R-I + II (for bacteria) or UniTag1-518f and UniTag2-905r (for archaea) and 20 ng of sample DNA. The cycling conditions for the first step consisted of an initial denaturation at 98°C for 30 s; 25 cycles of denaturation at 98°C for 10 s, annealing at 56°C (for bacteria) or 60°C (for archaea) for 20 s, and elongation at 72°C for 20 s; with a final extension at 72°C for 10 min.

The second step PCR was performed in a total volume of 100 μL containing: 1× HF buffer, 2 μL of dNTP Mix, 1 U of Phusion® Hot Start II High-Fidelity DNA polymerase (2 U/μL) and 500 nM of a forward and reverse primer targeting the UniTag1 and UniTag2 sequences, respectively, that were each appended with an 8 nt sample specific barcode at the 5’ end of the respective primer. The cycling conditions of the second step for both bacteria and archaea consisted of an initial denaturation at 98°C for 30 s followed by 5 cycles of: 98°C for 10 s, 52°C for 20 s and 72°C for 20 s, and a final extension at 72°C for 10 min. Incorporation of the sample specific barcodes, yielding a PCR product of ~350 bp and ~385 bp for bacteria and archaea, respectively, was confirmed by agarose gel electrophoresis. Control PCR reactions were performed alongside each separate amplification with no addition of template, and consistently yielded no product.

PCR products were then purified using HighPrep^TM^ (MagBio Europe Ltd, Kent, United Kingdom) and quantified using a Qubit fluorometer in combination with the dsDNA BR Assay Kit (Invitrogen, Carlsbad, USA). Purified PCR products were mixed in equimolar amounts into pools together with defined synthetic mock communities that allowed assessment of potential technical biases [23]. Library preparation and sequencing were then outsourced to GATC-Biotech, Konstanz, Germany (now part of Eurofins Genomics Germany GmbH). Library preparation was performed with an optimized protocol and standard Illumina adapter sequences. Sequencing was performed with Illumina MiSeq (read mode 2 x 150bp) for bacterial barcoded amplicons and with Illumina HiSeq Rapid Run (2x150 bp) for archaeal barcoded amplicons.

The 16S rRNA amplicon sequencing raw data for the bacterial and archaeal composition analysis has been deposited as one study in European Nucleotide Archive (ENA) under accession number PRJEB24373.

### Sequence data quality control and processing

The 16S rRNA gene sequencing data was analyzed using NG-Tax 2.0 [24], which executes four major tasks: demultiplexing and amplicon read cleaning, OTU-picking, denoising and taxonomic assignment. NG-Tax 2.0 defines OTUs using an open reference approach, and OTUs are defined as unique sequences that are above a user-defined minimum abundance threshold. NG-Tax 2.0 was run with the following default settings: 70 nt read length (i.e., 140 nt in total due to being paired-end data which was not merged), ratio OTU abundance 2.0, classify ratio 0.8, minimum percentage threshold 0.1%, identity level 100% and error correction of one mismatch (98.5%). Paired-end libraries were filtered to contain only read pairs with perfectly matching barcodes, and those barcodes were used to demultiplex reads by sample. The chimera detection process used the following condition: if the forward and reverse read of the OTU was identical to two different OTUs in the same sample and the abundance of the matched OTUs were at least twice of the abundance, then the OTU was marked as chimeric. Taxonomy was assigned to OTUs in NG-Tax 2.0 as previously described [24] using the 128 version of the SILVA 16S rRNA gene reference database [25].

### Data analysis and visualization

Parameters related to ruminal fermentation and microbial concentrations were analyzed using the MIXED procedure in SAS (edition 9.3, SAS Institute Inc., Cary, USA). The model included diet, time, and diet × time as fixed effects, and block and cow as random effects. The parameters were subjected to repeated measures ANOVA to take into account the repeated sampling from the same animal. Post-hoc analyses were carried out using the Tukey-Kramer test for pairwise comparisons. Significance of fixed effects was declared at P ≤ 0.05, and trends at 0.05 < P < 0.10.

The correlation between CH_4_ emissions and archaeal concentrations per time point (i.e., day 10 and day 17) was determined using the CORR procedure in SAS (edition 9.3, SAS Institute Inc., Cary, USA) with CH_4_ production (g/d), CH_4_ yield (g/kg DMI), archaeal concentrations (log_10_ 16S rRNA gene copies/mL of rumen fluid) and the archaea to bacteria ratio as variables. The CH_4_ data used was a subset of the data previously published [10], as only the data from the 12 cows in the present study was used. In terms of the subset of the CH_4_ yield (g/kg DMI) data, the mean values (± standard deviation) for the different diets were: 24.0 ± 0.34 for GS100; 24.7 ± 0.14 for GS67; 23.4 ± 0.44 for GS33 and 22.1 ± 0.48 for GS0. The data subset mean values (± standard deviation) of CH_4_ production (g/d) for the different diets were: 364 ± 18.0 for GS100; 393 ± 19.1 for GS67; 385 ± 3.2 for GS33, and 376 ± 10.5 for GS0.

Microbiota analysis was performed in R (version 3.4.0) [26]. Alpha diversity of the 16S rRNA gene data was estimated with the phylogenetic diversity (PD) index. Normality of the bacterial and archaeal PD dataset was assessed using the Shapira-Wilk’s normality method, with a P-value > 0.05 confirming normal distribution. Consequently, effects of diet, and diet × time were assessed using a Kruskal-Wallis test on the PD dataset while the time effect on the PD was assessed by a paired T-test. To assess the beta diversity in bacterial and archaeal communities in the cows across all four experimental diets, unweighted and weighted UniFrac distances were used to perform principal co-ordinate analysis (PCoA). Using the adonis function in vegan [27], PERMANOVA was used to test for significance of sample groupings with respect to diet, time and diet × time [28]. The R packages used to perform and visualize the community based analysis in RStudio were: ape version 5.2, vegan version 2.5–3, microbiome version 1.5.28, phyloseq version 1.24.2, picante version 1.7. and ggplot2 version 3.1.0 [27,29–31]. Further details of the bacterial and archaeal analysis performed in R is available as R markdown files: https://github.com/mibwurrepo/Vaidya_JD_2019_RumenDietMicrobiome.

Constrained partial redundancy analysis (RDA) of bacterial and archaeal 16S rRNA gene sequence data was performed to assess the relationship between genus level phylogenetic groupings and explanatory variables diet with covariance time, time with covariance diet and diet × time, using Canoco 5 [32]. Significance of explanatory variables was tested using a Monte Carlo permutation test with a total of 999 permutations.

All P-values for beta-diversity statistical analysis were corrected for multiple testing using False discovery rate (FDR) correction. Significant effects were declared at P ≤ 0.05, and trends at 0.05 < P < 0.10.

## Results

### Ruminal fermentation characteristics

Ruminal fermentation products are presented in Table 2. The molar proportions of butyrate tended to be affected by diet (P = 0.065). Total VFA tended to be affected by time (P = 0.076) and the molar proportions of isovalerate were affected by time (P = 0.002), being lower on day 17 than on day 10.

**Table 2:** Ruminal fermentation products and microbial concentrations measured in dairy cows after 10 or 17 days of feeding different proportions of grass silage (GS) and/or maize silage (MS)^1^.

Item	Diet (D)				Time (T)		SEM^2^	P-value		
	GS100	GS67	GS33	GS0	10	17		D	T	D x T
**Total VFA (mM)**	105.4	97.3	99.1	103.5	96.8	105.9	6.88	0.571	0.076	0.877
**VFA (% of Total)**										
Acetate	64.9	65.1	65.2	62.9	64.6	64.5	1.20	0.320	0.859	0.939
Propionate	20.0	19.3	18.8	17.7	19.2	18.8	0.76	0.101	0.326	0.850
Butyrate	13.8	14.1	14.5	17.6	14.1	16.5	1.09	0.065	0.168	0.917
Isovalerate	1.3	1.5	1.4	1.8	1.7^x^	1.3^y^	0.22	0.414	0.002	0.384
**Microbial Conc.**^3^										
Bacteria	10.9^a^	10.8^a^	10.8^a^	10.5^b^	10.6^x^	10.9^y^	0.10	0.026	0.008	0.762
Archaea	8.2	8.2	8.2	8.0	8.1^x^	8.2^y^	0.20	0.174	0.022	0.492

^1^ Mean values with a different superscript indicate a significant difference in diet (^a,b^), or time (^x,y^).

^2^ SEM refers to standard error of the mean.

^3^ Microbial concentration values are expressed as Log_10_ (16S rRNA gene copies/ml rumen fluid).

### Quantification of bacteria and archaea

The rumen bacterial and archaeal concentrations are presented in Table 2. The bacterial concentrations were affected by diet (P = 0.026) and time (P = 0.008). The bacterial concentrations were lower for the GS0 diet compared with the other three diets (GS100, GS67, and GS33), and the bacterial concentrations were higher on day 17 compared with day 10. The archaeal concentrations were only affected by time (P = 0.022), with the archaeal concentration being higher on day 17 compared with day 10.

The subset of the previously published CH_4_ data of van Gastelen et al [10] that related to the animals in the present study was used to assess if any correlations existed between microbial concentrations and CH_4_ production or CH_4_ yield. When considering all 12 animals (i.e., not taking the effects of the diet fed into account), the archaeal concentration (log_10_ 16S copies/mL of rumen fluid) measured on day 10 was not related to CH_4_ production (g/d) (P = 0.134), but was related to CH_4_ yield (g/kg DMI) (r = 0.69, P = 0.019). No relationships were found between the archaeal concentration measured on day 17 and either CH_4_ production or yield (P > 0.760). Additionally, the archaea to bacteria ratio measured on day 10 was not related to either CH_4_ production or yield (P > 0.329). Also, the archaea to bacteria ratio measured on day 17 was not related to CH_4_ yield (P = 0.712), but was related to CH_4_ production (r = -0.77, P = 0.005).

### Changes in rumen bacterial community composition

**Fig 1:**
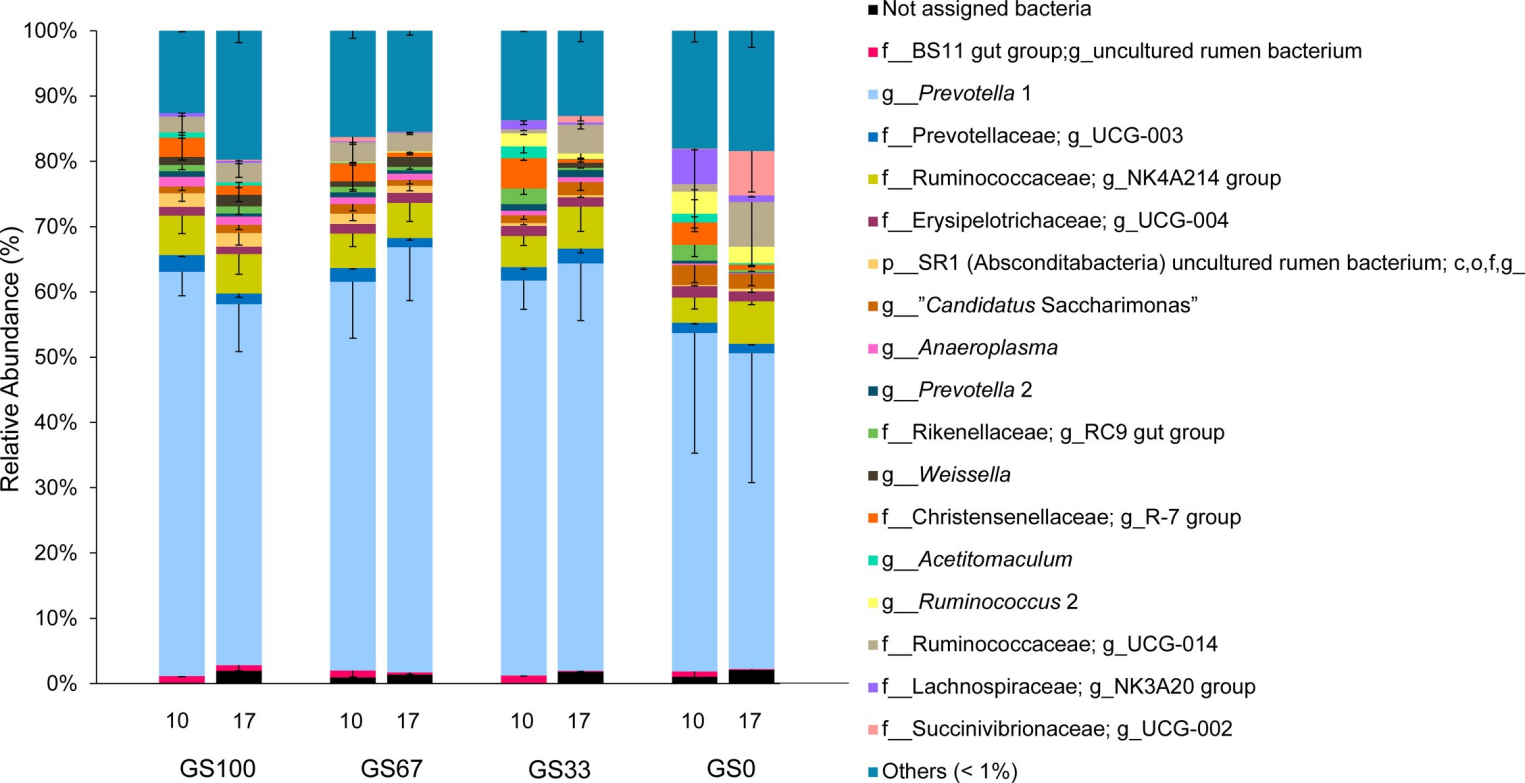
Relative abundance of major (>1%) bacterial genus level groups in rumen fluid samples from dairy cows. Genus level groups that were <1% (in at least one sample) are summed as ‘Others’. Sample codes indicate the number of days that the diet had been fed (10 or 17) and the different grass and maize silage proportions in the diet. Bars represent mean values (n = 3), and error bars the standard deviation. ‘c,o,f,g_” indicates that the corresponding taxonomic ranks (i.e., class (c), order (o), family (f) or genus (g)) could not be annotated.

**Fig 2:**
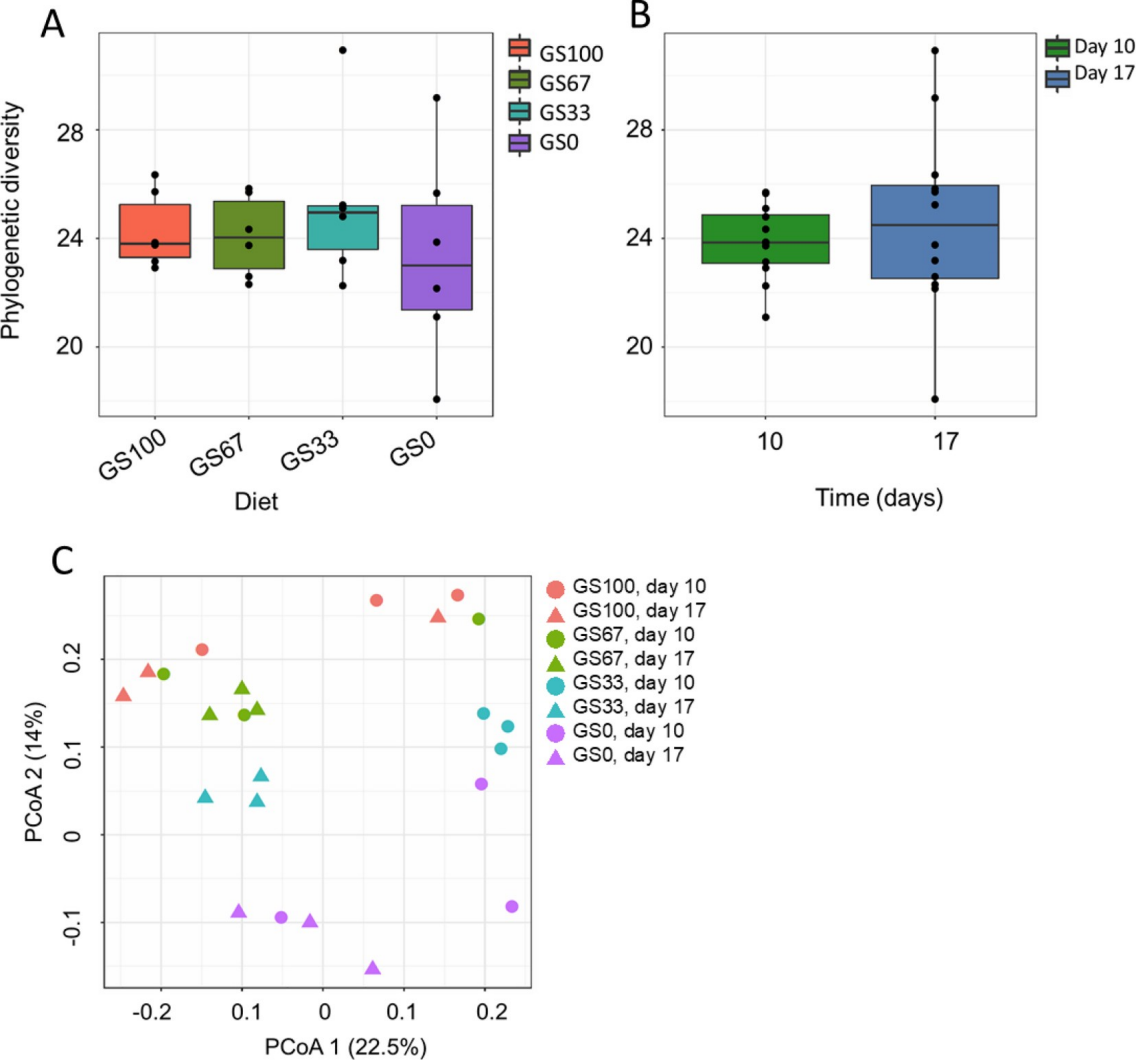
Alpha and beta diversity analysis of rumen bacterial communities. Phylogenetic diversity (PD) of rumen bacterial communities associated to different ratios of (A) grass and maize silage and (B) different time points. Sample codes indicate the number of days that the diet had been fed (10 or 17) and the different grass and maize silage proportions in the diet, for example GS100 is 100% grass silage. Principal co-ordinate analysis (PCoA) analysis of bacterial community composition in rumen fluid samples (n = 24) using (C) the unweighted UniFrac distance metric. The percentage of variation explained is indicated on the respective axes.

In total, 1,845 bacterial OTU were detected in the dataset. At the genus level, *Prevotella* 1 (58.1% ± 0.06) dominated in all the samples. The next most predominant genus level group was the NK4A214 group belonging to the Ruminococcaceae family (5.5% ± 0.01), with all other genus level groupings being more minor in abundance (Fig 1). With respect to alpha diversity, the PD values were confirmed to have a normal sample distribution (P = 0.196). There was no significant effect of diet (P = 0.500; Fig 2A), time (P = 0.761; Fig 2B) or diet × time (P = 0.697) on bacterial PD.

With respect to beta diversity, PCoA analysis at the OTU-level, was performed using unweighted UniFrac distances (Fig 2C). A significant clustering of the bacterial RF samples with diet (P = 0.006, R^2^ = 0.212) and time (P = 0.005, R^2^ = 0.094) was found, but no diet × time interaction (P = 0.763, R^2^ = 0.095). With respect to diet, in general, the profiles of GS100 diet fed animals clustered at the top of PCoA axis 2, the GS0 diet fed animals clustered at the bottom of the PCoA axis 2, and the mixed proportion diets (GS67 and GS33) were situated between the two extreme diets (Fig 2C). With respect to time, samples taken at day 17 and day 10 were generally separated along PCoA axis 1 (Fig 2C). In the corresponding weighted UniFrac analysis, there was a tendency for an effect of diet (P = 0.062, R^2^ = 0.213), whereas no time effect (P = 0.139, R^2^ = 0.071) or diet × time interaction (P = 0.911, R^2^ = 0.068) was seen (S1 Fig).

**Fig 3:**
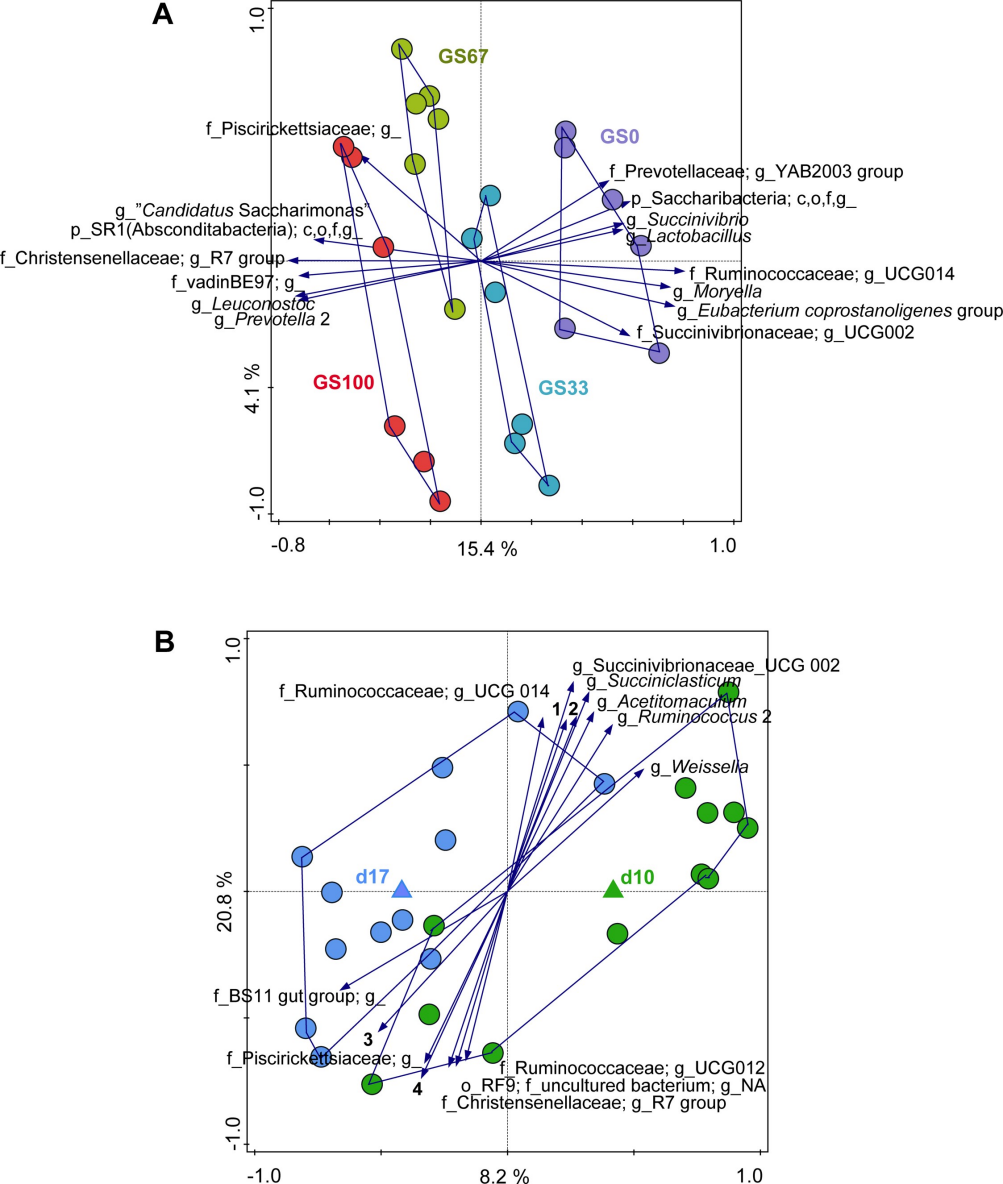
Redundancy analysis of rumen bacterial communities. Redundancy analysis triplots of partial RDA showing the relationship between the top 15 genus level phylogenetic groupings of the OTUs for which the variance is best explained by the constrained axes. Arrow length indicates the variance that can be explained by (A) diet with covariate time and (B) time with covariate diet. The axes are labelled with the amount of variation they represent. With (B), only the first axis is constrained and the explained variance is higher for the second (i.e., the first unconstrained) axis. Arrow labels indicate the taxonomic identification of genus level phylogenetic groupings of OTUs, with the level of the taxonomic annotation indicated (i.e., phylum (p), class (c), order (o), family (f), or genus (g)). “c,o,f,g_” and “g_” indicates that the corresponding taxonomic ranks could not be annotated due to no matches to any taxonomic lines in the Silva database. For example, “f_BS11 gut group; g_” could only be annotated to the family level. “g_NA” indicates that whilst there were hits with the Silva database at the genus level, the taxonomy could not be reliably assigned. Sample codes for the means indicate either (A) different grass and maize silage proportions in the diet (i.e., GS100 is 100% grass silage) or (B) the number of days that the diet had been fed (i.e., d10 is 10 days of feeding). Due to space restrictions on the plot in (B), the labels for four of the arrows are indicated here as follows: 1 (g_*Butyrivibrio* 2); 2 (g_*Eubacterium coprostanoligenes* group); 3 (o_RF9; f_uncultured bacterium; g_); 4 (o_RF9; f_uncultured rumen bacterium; g_NA).

In order to assess the contribution of diet, time and diet × time interaction to the observed variation in the bacterial community composition at the genus level, constrained RDA analyses were performed. Diet was associated with 24.5% of the total variation in the bacterial community (P = 0.002) when time was used as a covariate. In the RDA triplot (Fig 3A), samples of animals fed the different experimental diets separated along the first canonical axis according to the decreasing proportion of GS (i.e., from left to right). Several of the genus level groups had highest relative abundance with one of the extreme diets (i.e., either GS100 or GS0). The following genus level groups were positively associated with the GS0 diet: the UCG-002 group belonging to the Succinivibrionaceae, a genus level group containing *Eubacterium coprostanoligenes*, *Moryella*, the UCG-014 group belonging to the Ruminococcaceae family, *Lactobacillus*, *Succinivibrio*, the YAB-2003 group belonging to the Prevotellaceae family and OTUs that could only be reliably annotated to the phylum Saccharibacteria. In contrast, the genus level groups *Prevotella* 2, *Leuconostoc*, and “*Candidatus* Saccharimonas” were positively associated with the GS100 diet, along with several OTUs that could only be annotated to the family (Piscirickettsiaceae, vadin BE-97) or phylum level (SR1(Absconditabacteria)).

Partial RDA analysis with diet as covariate showed that time had an effect (P = 0.015) on the bacterial composition and explanatory variables accounted for 10.56% of the residual variation in the bacterial community (Fig 3B). Although time appears to separate on the first canonical axis, there was no clear association of any bacterial genus level groups to either of the days. The full RDA analysis showed that there was a diet × time interaction (P = 0.011) which accounted for 39.04% of the total variation in the bacterial community (S2 Fig). Generally, the diets separated along the first canonical axis (GS decreasing from left to right) and time on the second canonical axis (day 10 to the top and day 17 to the bottom). The extent of the differences between days 10 and 17 varied with diet, with the differences being smallest for GS67 and largest for GS33.

### Changes in rumen archaea community composition

**Fig 4:**
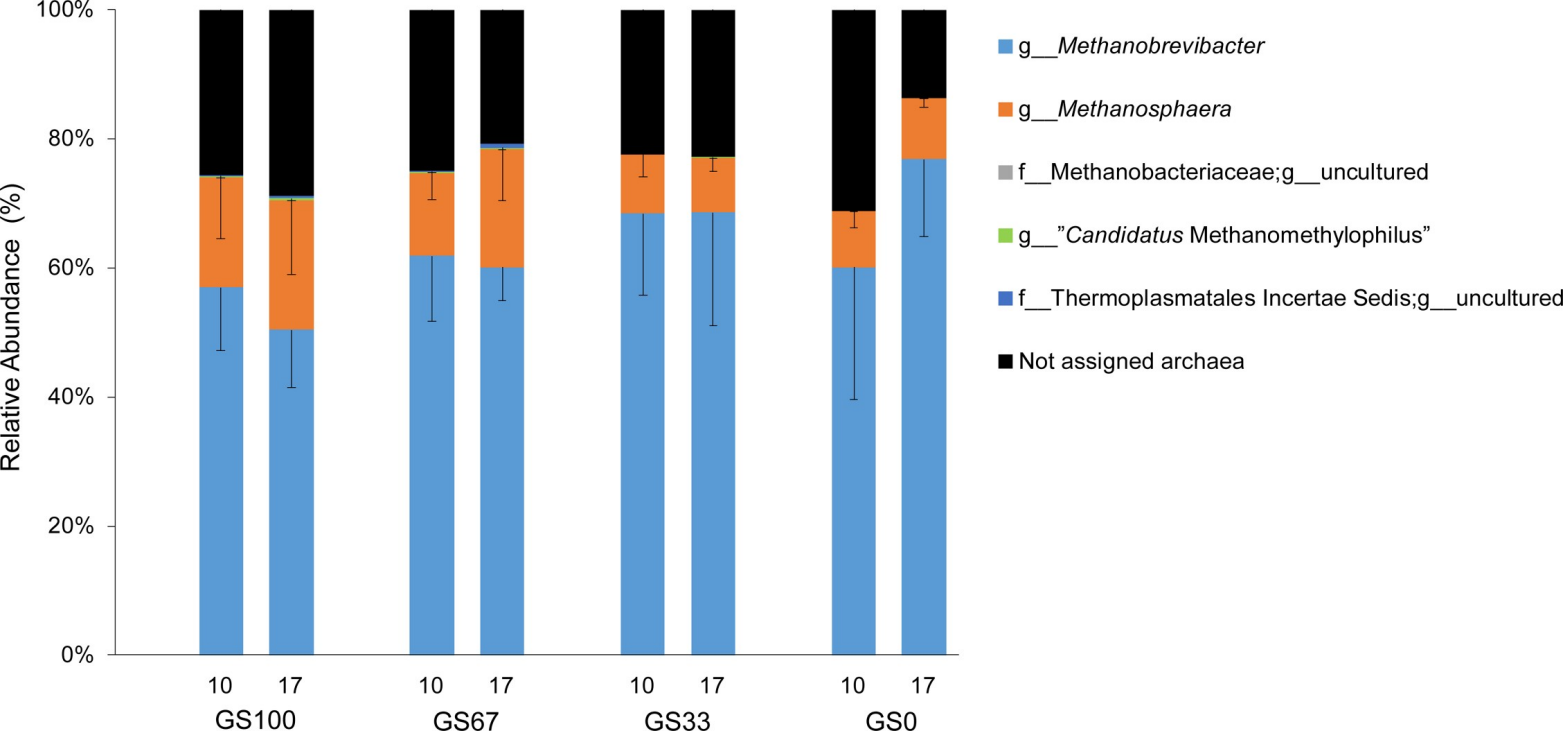
Relative abundance of all archaeal genus level groups in rumen fluid samples from dairy cows. Sample codes indicate the number of days that the diet had been fed (10 or 17) and the different grass and maize silage proportions in the diet, for example GS100 is 100% grass silage. Bars represent sample means from cows fed the same diet (n = 3), and error bars represent their standard deviation.

**Fig 5:**
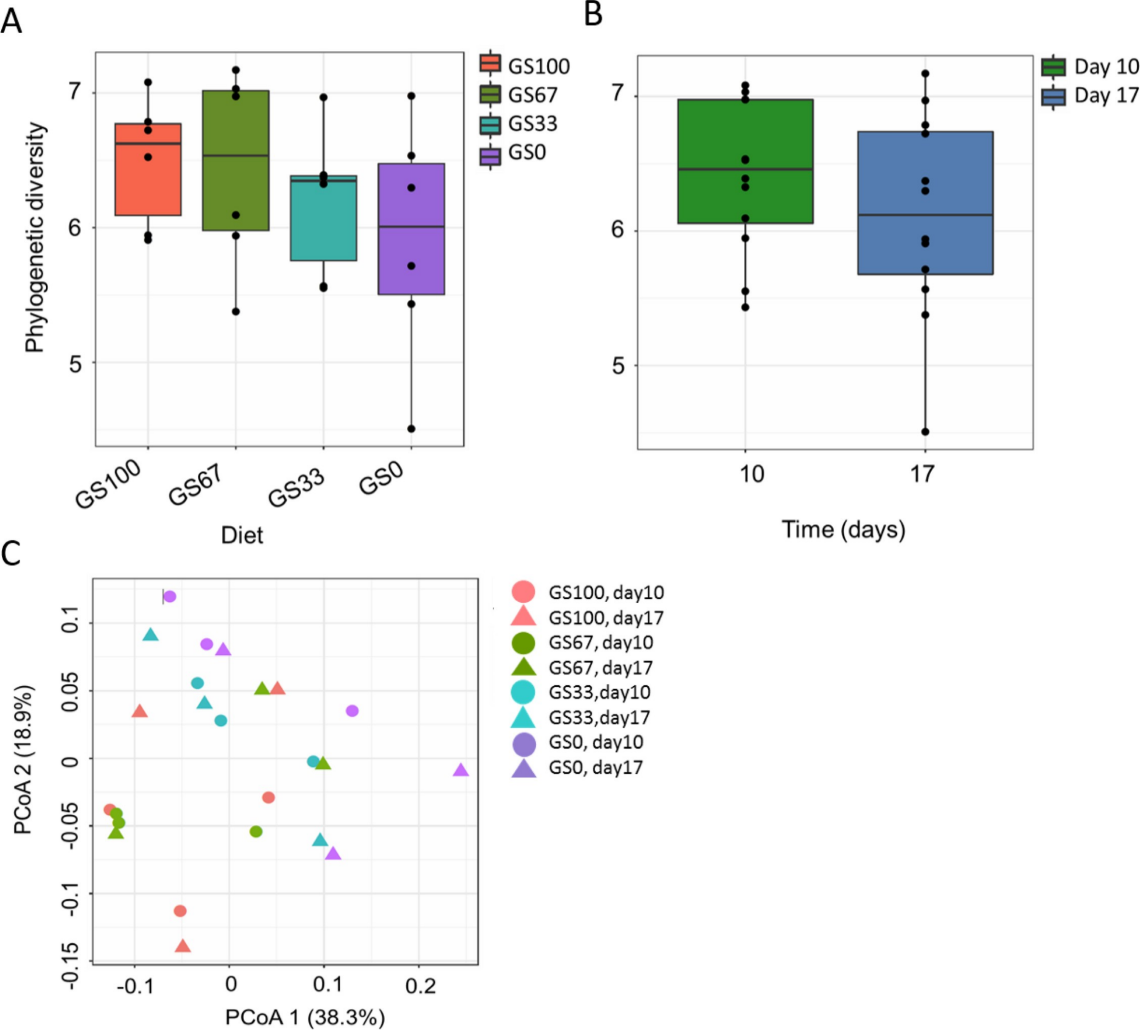
Alpha and beta diversity analysis of rumen archaeal communities. Phylogenetic diversity (PD) of archaeal communities associated to (A) different ratios of grass and maize silage and (B) different time points. Sample codes indicate the number of days that the diet had been fed (10 or 17) and the different grass and maize silage proportions in the diet, for example GS100 is 100% grass silage. Principal co-ordinate analysis **(**PCoA) analysis of archaeal community composition from rumen fluid samples (n = 24) using (C) the unweighted UniFrac distance metric. The percentage of variation explained is indicated on the respective axes.

Annotation of the 326 detected OTUs showed that *Methanobrevibacter* was the predominant archaeal genus followed by *Methanosphaera* (Fig 4). Archaeal PD values were confirmed to have a normal distribution (P = 0.189). There was no effect of diet (P = 0.300; Fig 5A), time (P = 0.525; Fig 5B) or diet × time (P = 0.272) on the archaeal PD. With unweighted UniFrac distances of the archaeal community (Fig 5C), a tendency for a diet effect was observed (P = 0.075, R^2^ = 0.196), but no effect of time (P = 0.505, R^2^ = 0.036) or diet × time interaction (P = 0.582, R^2^ = 0.109). In the weighted UniFrac analysis, no effects of diet (P = 0.862, R^2^ = 0.044), time (P = 0.430, R^2^ = 0.034) or diet × time interaction (P = 0.534, R^2^ = 0.111) were observed (S3 Fig).

**Fig 6:**
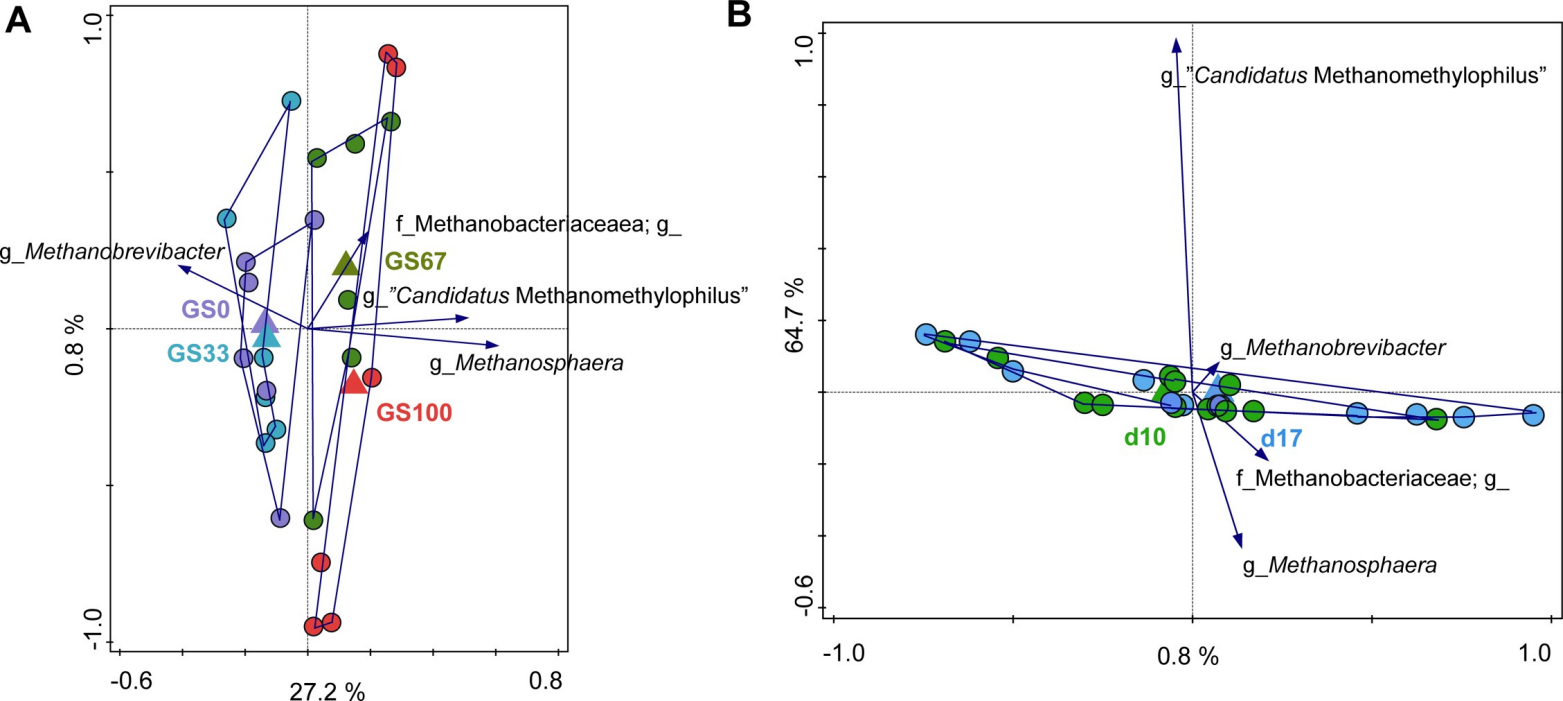
Redundancy analysis of rumen archaeal communities. Redundancy analysis triplots of partial RDA showing the relationship between the top 15 genus level phylogenetic groupings of the OTUs for which the variance is best explained by the constrained axes. Arrow length indicates the variance that can be explained by (A) diet with covariate time and (B) time with covariate diet. The explanation of the plots and the labels for the taxa and samples are as previously described in Fig 3.

The contribution of diet, time and diet × time interaction to variation in the archaeal community composition at the genus level was assessed using partial RDA analyses. Diet was associated with 28.34% of the residual variation in the dataset (P = 0.032) when time was used as covariate (Fig 6A). Almost all of this variance was represented by the first canonical axis, where both GS100 and GS67 separated from GS33 and GS0 (with the latter two not different from one another). *Methanobrevibacter* showed a positive association with the GS0 and GS33 diets, *Methanosphaera* appeared to be most positively associated with the GS100 diet, and a non-annotated genus that belonged to the family Methanobacteriaceae was most positively associated with the GS67 diet. RDA analysis showed that time did not affect the archaeal community composition (P = 0.284) when diet was used as covariate, and only accounted for 1.24% of the residual variation (Fig 6B). No diet × time interaction was observed (P = 0.312, data not shown).

## Discussion

Replacing GS with MS in the diet of dairy cows was previously shown to result in a beneficial 11% decrease in CH_4_ emissions [10]. The objective of the current study was to characterize the rumen bacterial and archaeal communities in dairy cows fed different ratios of MS and GS in order to advance our understanding of how nutritional driven differences in CH_4_ yield and VFA relate to underlying changes in the rumen microbiome.

### Effect of diet and time on VFA production

The results of the rumen fermentation products (i.e., total VFA and molar proportions of individual VFA) in this study are largely in agreement with the results reported previously by Van Gastelen et al. [10]. Both the present study and Van Gastelen et al. [10] report no effect of diet on total VFA and the molar proportions of acetate and propionate. In the present study, however, the molar proportions of butyrate tended to be affected by diet, whereas Van Gastelen et al. [10] reported a linear increase in molar proportions of butyrate upon increasing the level of MS at the expense of GS. Additionally, Van Gastelen et al. [10] reported that isovalerate tended to be affected by diet, whereas in the current study no diet effect on isovalerate was found. These differences between the two studies are likely to be associated with the differences in frequency and timing of sampling.

In the present study, the RF samples were collected on day 10 (before the climate respiration chamber phase) and day 17 (after the climate respiration chamber phase), but only 4 h after morning feeding. In the study of Van Gastelen et al. [10], the RF samples were collected 1 h before and 1, 2, 4, 6 and 8 h after morning feeding on days 10 and 11 (i.e., before the climate respiration chamber phase). It is well documented that there are significant temporal variations in the molar proportions of individual VFA after feeding [4]. Furthermore, differences in VFA sample preparation and analysis between the two studies may have affected the results. Hence, direct comparison between the VFA results of the present study with those reported by Van Gastelen et al. [10] is hampered.

Isovalerate molar proportions were affected by time in the present study, with a decrease of isovalerate from day 10 to day 17. Usually, isovalerate is associated with microbial protein synthesis and fermentation of plant cell walls [33]. The decrease in isovalerate molar proportions might suggest a reduction of the cellulolytic bacterial populations for which isovalerate is an essential growth factor, as has been previously demonstrated in pigs [34]. However, this seems unlikely, as no bacterial genus level groups could be clearly associated with the effect of time on the bacterial community composition (Fig 3B).

### Effect of diet and time on rumen microbial concentrations

Diet affected bacterial concentrations, which were lower for the GS0 diet compared with the other three diets. This is contrary to the expectation of having a higher bacterial concentration in the rumen liquid of animals fed the GS0 diet, because of the high concentration of starch which is a more easily fermented substrate for bacteria than the fiber in the GS containing diets [35]. The latter was also found by Lettat et al. [36], who reported increased bacterial concentrations upon completely replacing fiber-rich alfalfa with starch-rich MS when collecting rumen fluid 4 hours after morning feeding. In the present study, the bacterial concentration did not appear to be affected by the actual amount of MS fed, as only a decrease in bacterial concentrations in the absence of GS in the diet was observed. This suggests that the effect on bacterial concentrations might not be directly related to the amount of MS in the diet, but rather the absence of GS. The GS0 diet consisted only of MS, which is generally associated with a higher digestibility, a shorter residence time in the rumen, and a faster outflow of digesta. This may result in a faster outflow of microbial biomass from the rumen as well, offering a potential explanation for the results of the present study. There was no diet effect on archaeal concentrations which is in line with the study of Lettat et al. [36], who also did not observe any effect of diet on the methanogenic archaea. However, the numerically lower concentrations of archaea in the GS0 compared to the other GS containing diets is in line with the suggested faster outflow of microbial biomass of the rumen when GS is absent from the diet.

The observed increase in bacterial concentrations from day 10 to day 17 is suggestive of an increase in fermentation. However, total VFA only tended to be affected by time indicating the increase in bacterial concentration was not biologically significant. Also, archaeal concentrations were found to increase from day 10 to day 17. The reason for this increase is presumably linked to the similar change in the bacterial concentrations, as no significant change in archaeal community composition occurred with time (Fig 6B).

It seems intuitive that CH_4_ emissions should correspond to the number of archaea, as these are the only CH_4_-producing microorganisms present in the rumen. However, numerous studies have repeatedly failed to find such correlations between CH_4_ emission and archaeal concentration [37,38]. The results of this study also suggest that the relationship between archaeal concentrations and CH_4_ emission is not straight forward. The strong relationship found for archaeal concentrations and CH_4_ yield on day 10 was no longer found on day 17, whereas the opposite was observed for the archaea to bacteria ratio. The few studies that have found a relationship are based on single time-point measurements, which used either post-mortem rumen digesta samples [38] or a relatively large number of animals [39]. The biological significance of the positive correlation of archaeal concentrations with CH_4_ yield on d10 of this study are, however, not clear as the CH_4_ emission data from the study of Van Gastelen et al. [10] was measured only from days 13 to day 17. In order to gain more insight, longitudinal studies looking at CH_4_ emissions and microbial concentrations over the same time course are needed.

### Effect of diet and time on rumen bacterial composition

Diet did not affect bacterial alpha diversity, however, it did affect bacterial community composition (Fig 2). This effect was observed in the unweighted UniFrac PcoA analysis (Fig 2C) but not in the corresponding weighted analysis (S1 Fig). This suggests that the diet effect was mainly due to differences in the presence or absence of low abundant bacterial taxa, rather than differences in the relative abundance of predominant bacterial taxa. In both the unweighted PcoA and the RDA, the bacterial community composition showed a transition that was consistent with the decreasing amount of GS.

The higher amount of starch present in MS has been associated with increased relative abundances of members of the families Ruminococcaceae and Succinivibrionaceae [40,41], which is consistent with the positive association of UCG-002, *Succinivibrio* and UCG-014 with the GS0 fed cows in the present study. Low CH_4_ producing cows have been positively associated with lactate and succinate producing bacteria [42]. This is also consistent with the positive association of *Lactobacillus* (a lactate producer) and *Succinivibrio* (a succinate producer) in the GS0 diet, as this diet was also associated with the lowest CH_4_ emission in the study of Van Gastelen et al. [10]. A metagenomics study has linked increased abundance of Succinivibrionaceae with reduced CH_4_ emission in dairy cows [43]. This can be explained by the fact that Succinivibrionaceae compete with hydrogenotrophic methanogenic archaea for H_2_ and produce succinate, which is then converted to propionate [44]. *Moryella*, which was positively associated with the GS0 diet in our study, has also been reported as one of the dominant groups in a maize starch fed diet [45].

The relevance of the positive association of a genus level group that includes *Eubacterium coprostanoligenes* with the GS0 diet in the present study is not clear, as the characterized species *E*. *coprostanoligenes* does not hydrolyze starch [46]. Only a few other *Eubacterium* spp. From the rumen (i.e., *Eubacterium uniforme* and *Eubacterium xylanophilus*) have been previously associated with MS derived feeds [40]. *E*. *coprostanoligenes*, however, has until now only been associated with hydrolysis of cholesterol esters to produce coprostanol [47]. Species in the *Eubacterium* genus are saccharolytic and ammonia producing, playing a role in amino acid fermentation. As ammonia production in the rumen consumes H_2_ gas, *Eubacterium* spp. might play a role in decreasing CH_4_ emissions by depriving methanogens of H_2_ [13,48]. The relation between the decreasing amount of GS and the unclassified group belonging to the Saccharibacteria and the YAB-2003 group belonging to the Prevotellaceae is unclear due to the limited knowledge with respect to the physiology of these organisms.

Several genus level groups were positively associated with the GS100 diet. The positive association of *Prevotella* 2 with the GS100 diet (Fig 3A; which also indicates a negative association with GS0) suggests that *Prevotella* 2 plays a role in fiber degradation. This seems to be contrary to the findings of Henderson et al. [1], who reported that members of *Prevotella* were more abundant in animals fed diets containing concentrates (i.e., concentrate-fed animals and animals fed mixed diets) than in forage-fed animals, suggesting that these are probably major producers of propionate and the propionate-precursor succinate. *Prevotella* are most often one of the core members identified in the rumen microbial population irrespective of diet [1,49]. Based on the physiologies of cultured representatives, members of *Prevotella* genus are known for producing high levels of propionate from concentrate based diets. However, the review of Tapio et al. [50] highlighted that, as previously reported by Kittelmann et al. [51] and Danielsson et al. [42], some *Prevotella* OTUs were correlated with a high CH_4_ phenotype, whilst others were associated with a low CH_4_ phenotype. This suggests functional versatility within the *Prevotella* genus, which is perhaps not surprising considering some organisms within this genus are only distantly related to each other [52]. The positive association of *Leuconostoc* to GS100 observed in this study is harder to explain in the context of fermentation as *Leuconostoc* spp. are lactic acid bacteria that are typical inhabitants of silage, and fermentation of MS is generally faster than that of GS. Members of the Christensenellaceae family have been associated with degradation of fiber [53], in line with the positive association of this group with the GS100 diet.

The alpha diversity of the bacterial communities was affected by time, indicating that the community was still changing after 10 days of diet adaptation (Fig 2B). Indeed, this is also reflected in the finding that bacterial concentrations were found to be higher on day 17 compared to day 10. Whilst separation of day 10 and day 17 samples occurred in the RDA, no taxa were strongly associated with either of the days (Fig 3). Potential reasons for time related changes might be the differences in eating behavior or the time spent eating between the treatments [54]. It may also be possible that the rumen microbiota simply needs longer to stabilize following dietary change. A recent study reported that it took approximately nine weeks for the rumen bacterial community composition of beef steers to stabilize following dietary adaptation from a pasture based diet to a corn/silage based diet supplemented with monensin [55]. As such, Clemmons et al. [55] recommended adaptation or wash-out periods of at least eight weeks should be used in order to ensure stability of the rumen microbiota when performing nutritional intervention studies.

### Effect of diet and time on archaea

Our analysis did not show any significant differences in the archaeal alpha diversity in response to diet, time or diet × time (Fig 5A and 5B). Changes in feed fermentation products can induce changes in the methanogenic community structure, however, as only limited effects on the VFA occurred in this study it is perhaps not surprising that the weighted and unweighted UniFrac PCoA showed no significant diet effect on archaeal beta diversity (Fig 5C). However, significant diet effects were observed in the RDA analysis which was based on the relative abundances of the taxa at the genus rather than the OTU level. The relative abundance of *Methanobrevibacter* was positively associated with the GS0 diet, whereas *Methanosphaera* and “*Candidatus* Methanomethylophilus” were negatively associated with the GS0 diet. *Methanobrevibacter* and *Methanosphaera* were the two most dominant archaea found, and were present in all of the dietary treatments. *Methanobrevibacter* spp. are usually formate, H_2_ and CO_2_ dependent hydrogenotrophs while *Methanosphaera* spp. are H_2_ dependent methylotrophs [54]. The current findings suggest that diet dependent competition for H_2_ between *Methanobrevibacter* and *Methanosphaera* occurs within the rumen [54], indicating that the concentrations and partial pressures of H_2_ in the rumen are key factors affecting methanogenesis [3].

## Conclusions

In this study, we assessed changes in the rumen microbiota in response to dietary treatments differing in the roughage composition after 10 and 17 days of feeding. These changes were used to help understand corresponding differences in ruminal VFA and previously observed CH_4_ measurements. The bacterial community composition was affected by diet, time and diet × time, whereas the archaeal community composition was only affected by diet. Several bacterial and archaeal genus level groups could be associated with diet, but not with time. Analysis indicated the increased use of hydrogen by succinate and lactate producing bacteria is likely to at least partially explain the previously reported lower CH_4_ emissions from MS fed dairy cows relative to GS fed dairy cows. Furthermore, time had a significant effect on both bacterial and archaeal concentrations, and also bacterial community composition. This indicates that the rumen microbiome had not stabilized after 10 days of feeding the experimental diets.

## Supporting information

S1 Fig Principal co-ordinate analysis of rumen bacterial community composition. Samples (n = 24) were analyzed using the weighted UniFrac distance metric. Colors indicate the different grass and maize silage proportions in the diet (i.e., GS67 is 67% grass silage and 33% maize silage), and the symbol shapes indicate the number of days that the diet had been fed (10 or 17). The percentage of variation explained is indicated on the respective principal co-ordinate (PCoA) axes.
(TIF)

S2 Fig Redundancy analysis of diet × time on the bacterial and archaeal communities. A redundancy analysis triplot of bacterial communities are shown for the explanatory variable diet × time. The axes are labelled with the amount of variation they represent. Sample codes for the means (black filled symbols) indicate different grass and maize silage proportions in the diet (i.e., GS67 is 67% grass silage and 33% maize silage) and the number of days that the diet had been fed (d10 or d17).
(TIF)

S3 Fig Principal co-ordinate analysis of rumen archaeal community composition. Samples (n = 24) were analyzed using the weighted UniFrac distance metric. Colors indicate the different grass and maize silage proportions in the diet (i.e., GS67 is 67% grass silage and 33% maize silage), and the symbol shapes indicate the number of days that the diet had been fed (10 or 17). The percentage of variation explained is indicated on the respective principal co-ordinate (PCoA) axes.
(TIF)
