# Supplementary figures and images for: Characterization of dairy cow rumen bacterial and archaeal communities associated with grass silage and maize silage based diets
Source: PLoS One. 2020 Mar 2;15(3):e0229887. doi: 10.1371/journal.pone.0229887 (PMC7051090; doi:10.1371/journal.pone.0229887)

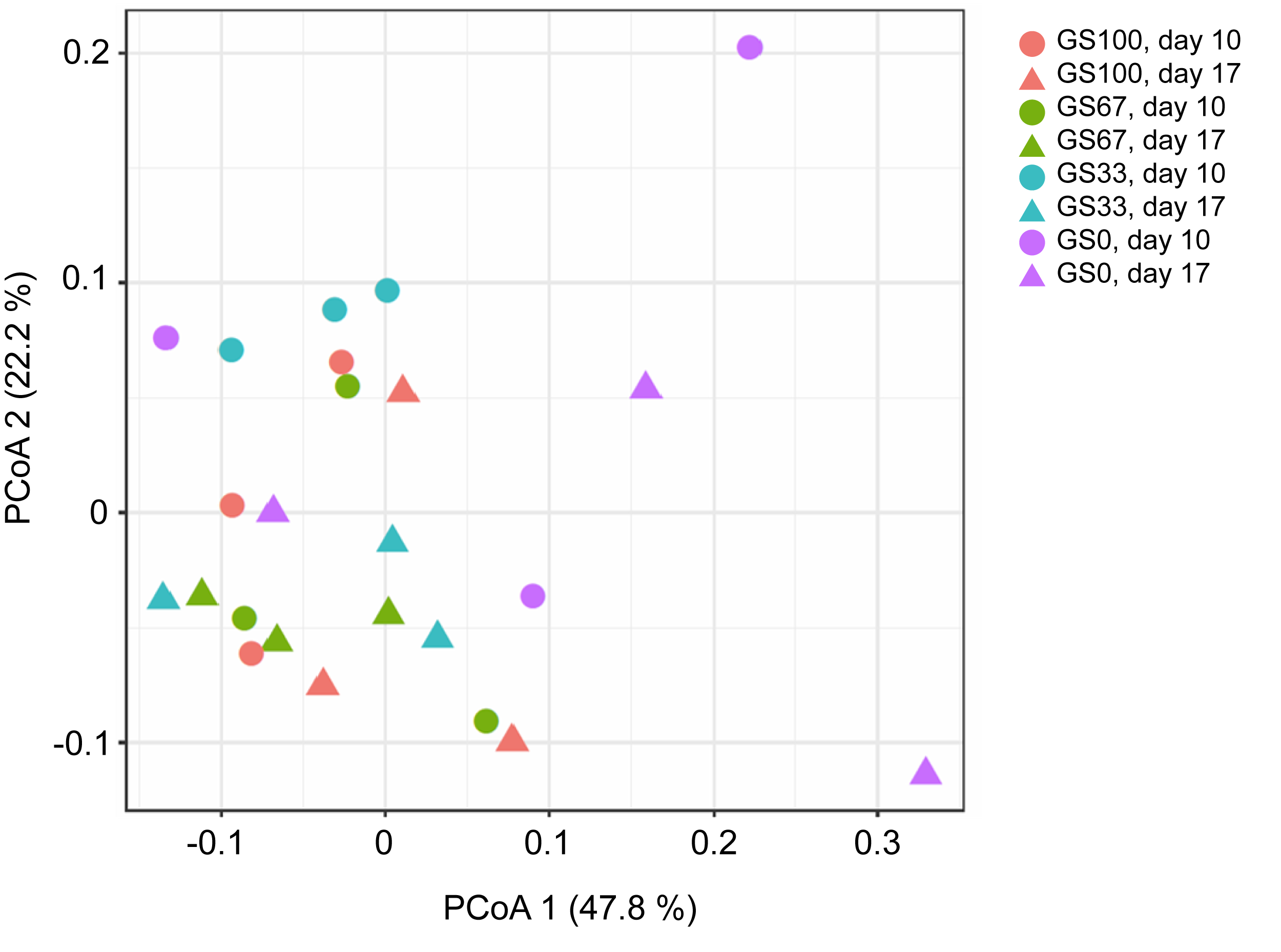

Supplement: S1 Fig — Samples (n = 24) were analyzed using the weighted UniFrac distance metric. Colors indicate the different grass and maize silage proportions in the diet (i.e., GS67 is 67% grass silage and 33% maize silage), and the symbol shapes indicate the number of days that the diet had been fed (10 or 17). The percentage of variation explained is indicated on the respective principal co-ordinate (PCoA) axes. (TIF) [file pone.0229887.s001.tif]

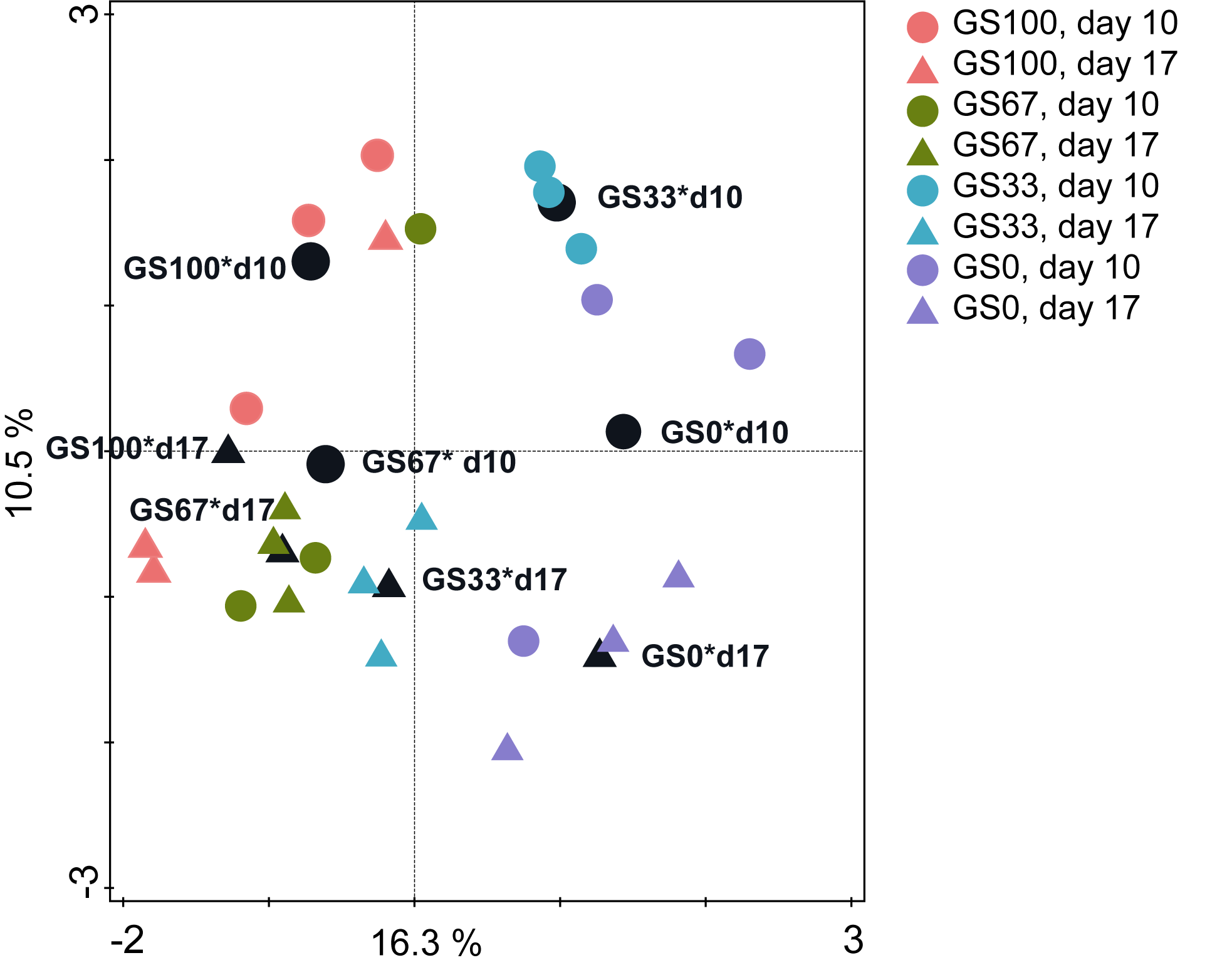

Supplement: S2 Fig — A redundancy analysis triplot of bacterial communities are shown for the explanatory variable diet × time. The axes are labelled with the amount of variation they represent. Sample codes for the means (black filled symbols) indicate different grass and maize silage proportions in the diet (i.e., GS67 is 67% grass silage and 33% maize silage) and the number of days that the diet had been fed (d10 or d17). (TIF) [file pone.0229887.s002.tif]

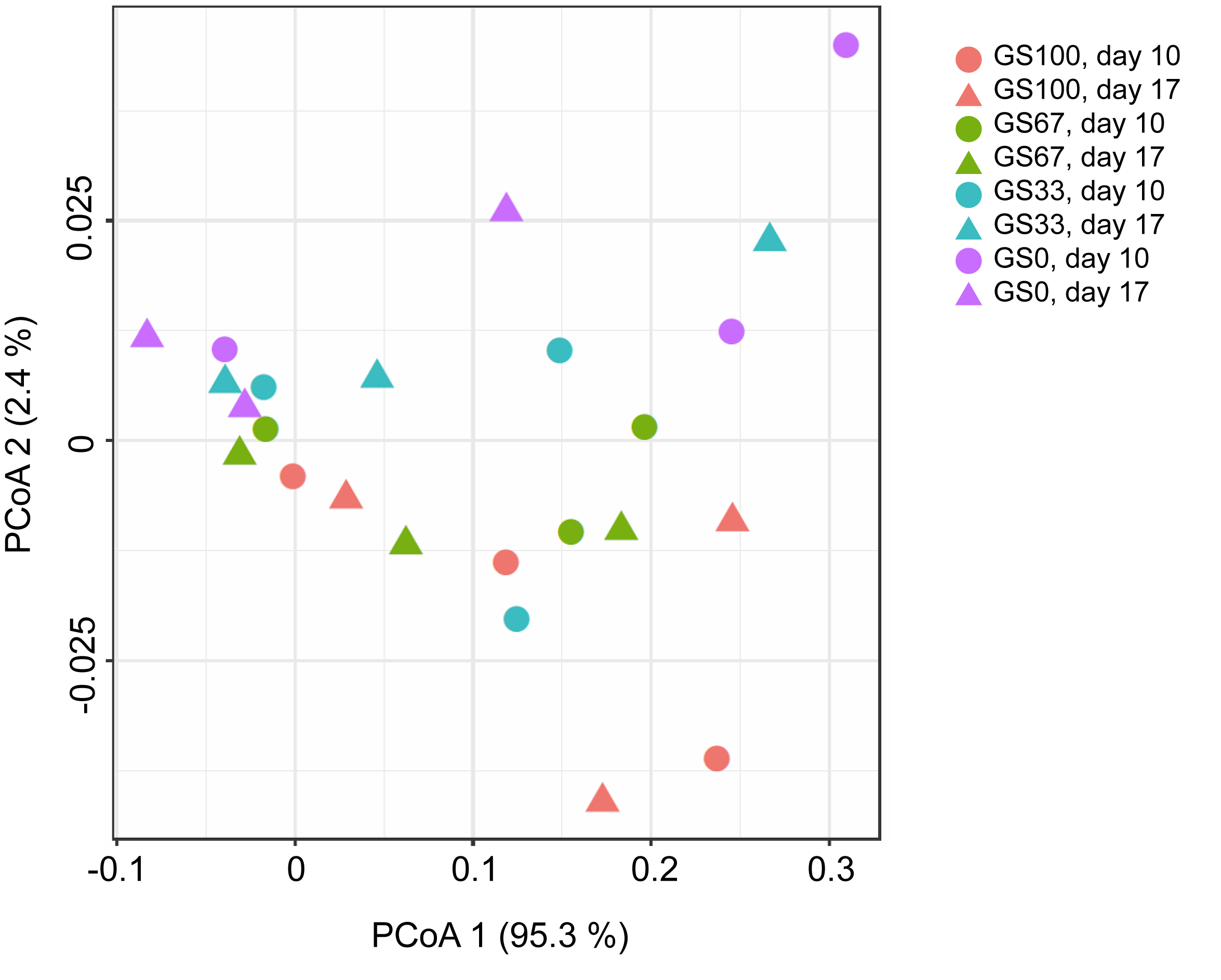

Supplement: S3 Fig — Samples (n = 24) were analyzed using the weighted UniFrac distance metric. Colors indicate the different grass and maize silage proportions in the diet (i.e., GS67 is 67% grass silage and 33% maize silage), and the symbol shapes indicate the number of days that the diet had been fed (10 or 17). The percentage of variation explained is indicated on the respective principal co-ordinate (PCoA) axes. (TIF) [file pone.0229887.s003.tif]
